# Supplementary material for: Efficient oral vaccination by bioengineering virus-like particles with protozoan surface proteins
Source: Nat Commun. 2019 Jan 21;10:361. doi: 10.1038/s41467-018-08265-9 (PMC6341118; doi:10.1038/s41467-018-08265-9)
Supplement: Supplementary file 1 — Supplementary Information [file 41467_2018_8265_MOESM1_ESM.pdf]

Efficient oral vaccination by bioengineering virus-like particles with protozoan surface proteins

Serradell, Rupil, *et al.*

Supplementary Figures

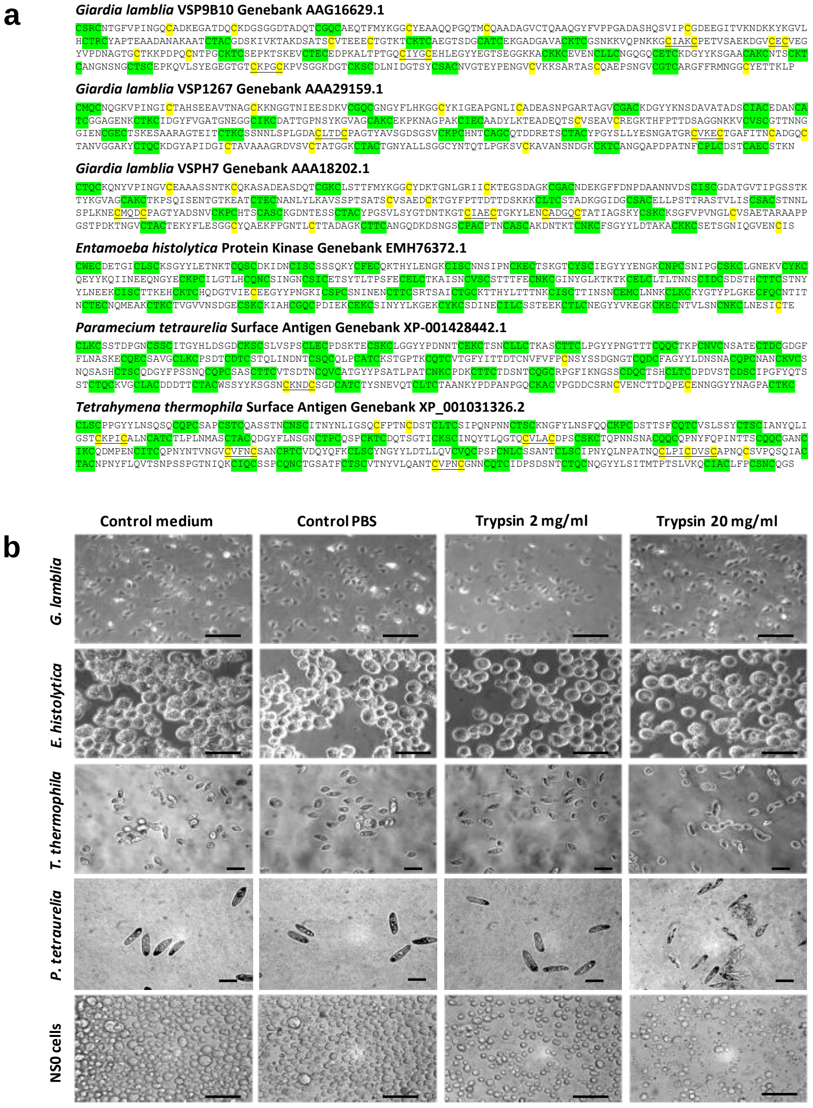

Supplementary Figure 1. Protease resistance of several protozoa cells with presence of proteins containing multiple CXXC motifs. **a**, Randomly selected portions of 450 amino acids from VSPs of the flagellated parasite *Giardia lamblia* (VSP1267, VSP9B10 and VSPH7) and VSP-like surface antigens of intestinal (*Entamoeba histolytica*, amoeboid parasite) as well as free-living microorganisms (*Tetrahymena thermophila* and *Paramecium tetraurelia*, ciliated) are shown. The CXXC motifs are highlighted in green. Cysteines not included in this motif are in yellow. In addition to the CXXC motif, in *Giardia* VSPs some cysteines are also included in CXC and CXXXC motifs (underlined) with some others not included in any motif, similar to VSP-like molecules from the other protozoa. **b**, Phase contrast images of trophozoites from *G. lamblia*, *E. histolytica*, *T. thermophila* and *P. tetraurelia*, and non-adherent mammalian cell (NS0) incubated during 90 min in different conditions. The bars represent 50 μm.

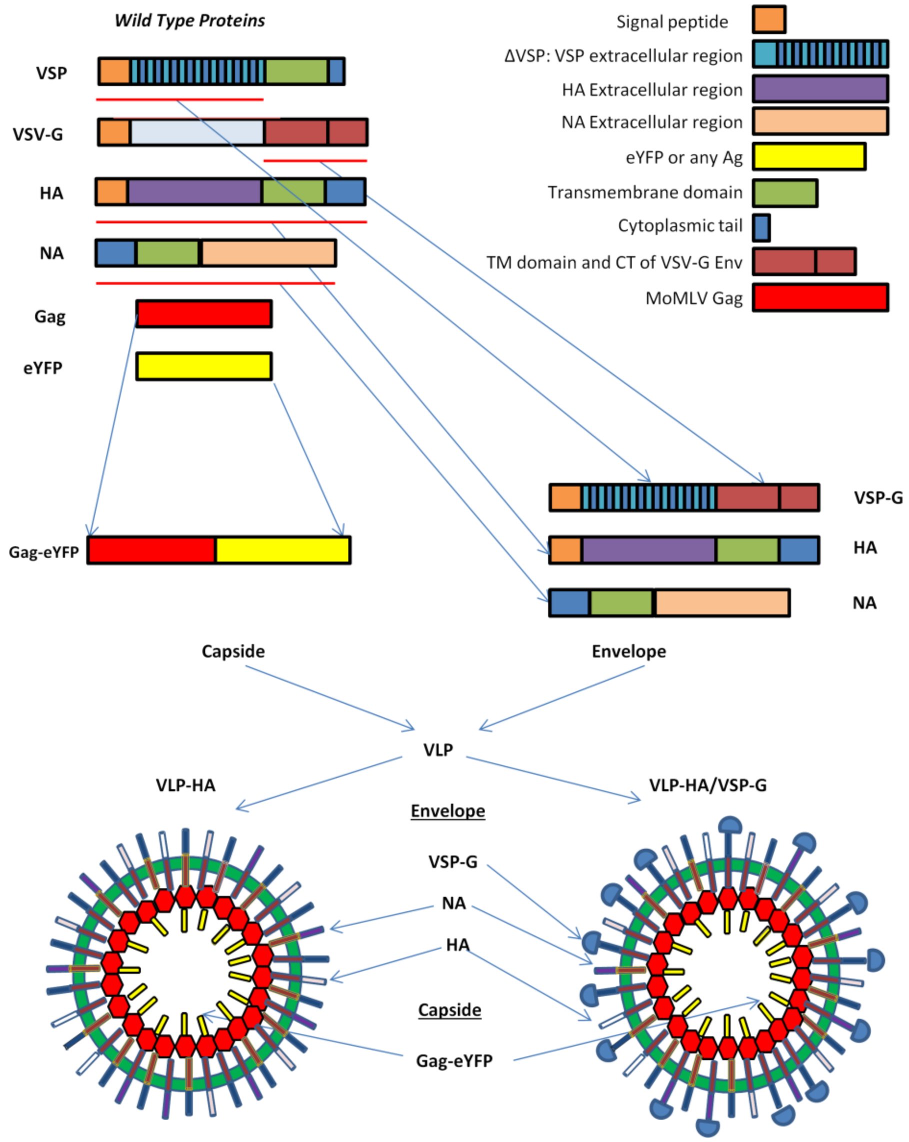

Supplementary Figure 2. Schematic representation of VLP design. Schematic representation of the wild type proteins and constructions generated to produce Virus-like Particles in eukaryotic cells. Colour-coded fragments are shown on the upper right. Transfection of cells with different plasmid combinations produces VLPs of different types, which are shown at the bottom as the final schematic representation of VLPs.

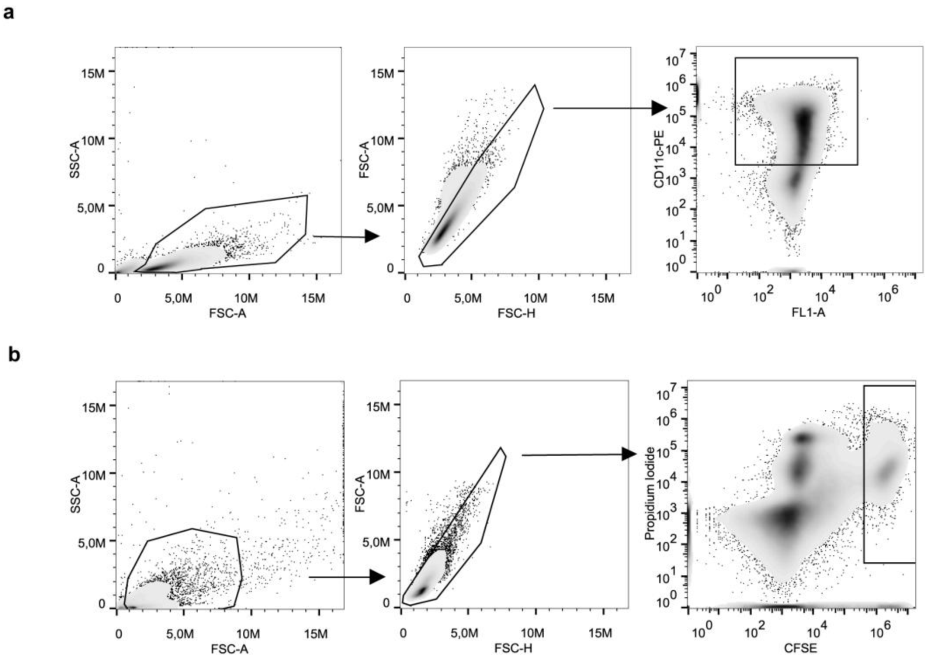

Supplementary Figure 3. Gating strategy dot blots. Target cells were gated based on forward and side scatter characteristics and then the singlet gate was performed. **a**, CD11c positive cells were selected on FL-2 (PE). **b**, The dead cells were analyzed on CFSE positive gate. SSC-A, side scatter area; FSC-H, forward scatter height; FSC-A, forward scatter area; CFSE, carboxyfluorescein succinimidyl ester.

Supplementary Figure 4. Uncropped Western Blots included in the corresponding figures of the main article.

Figure 1b

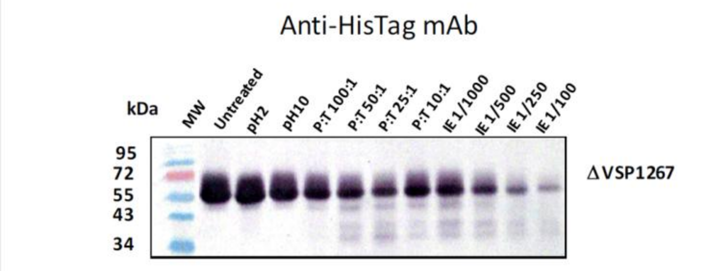

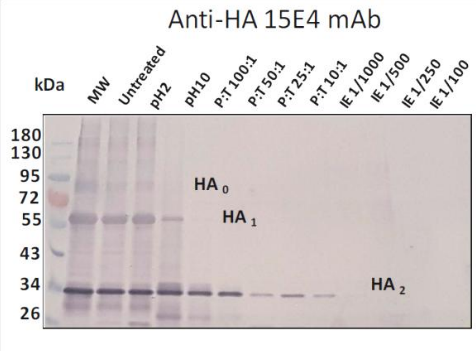

HA is synthesized as a precursor polypeptide HA_0 in the endoplasmic reticulum, where it is assembled into trimers. Then, via the Golgi network it is exported to the cell surface where it is cleaved by host proteases into two subunits: HA_1 and HA_2. The HA_1 subunit forms a globular head which contains the receptor binding site. It can be observed that this subunit presents a greater sensitivity to proteolysis than HA_2.

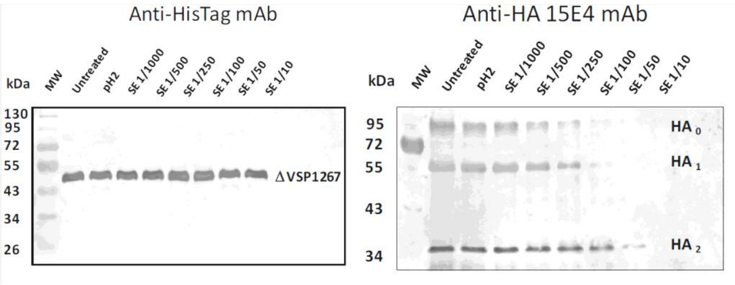

Figure 1c

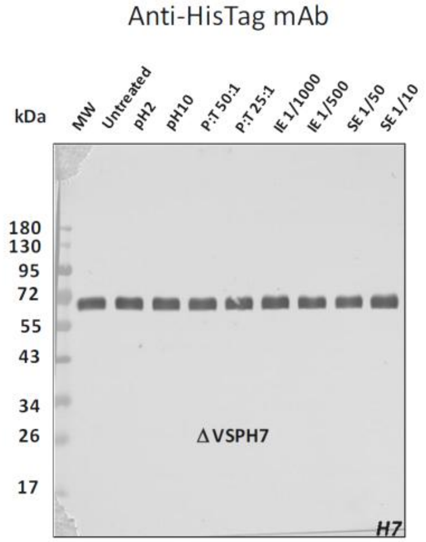
Anti-HisTag mAb
kDa
MW
Untreated
pH2
pH10
P:T 50:1
P:T 25:1
IE 1/1000
IE 1/500
SE 1/50
SE 1/10
180
130
95
72
55
43
34
26
17
ΔVSPH7
H7

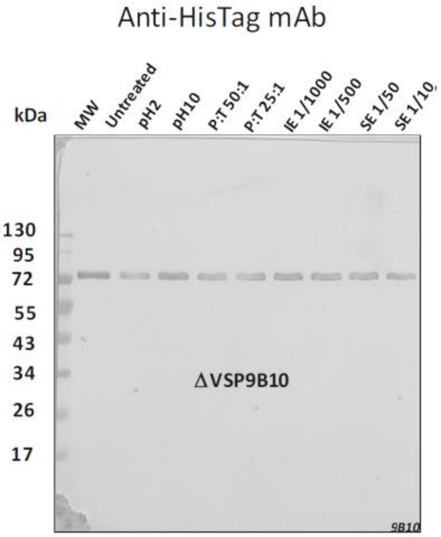
Anti-HisTag mAb
kDa
MW
Untreated
pH2
pH10
P:T 50:1
P:T 25:1
IE 1/1000
IE 1/500
SE 1/50
130
95
72
55
43
34
26
17
ΔVSP9B10
9B10

Figure 1d

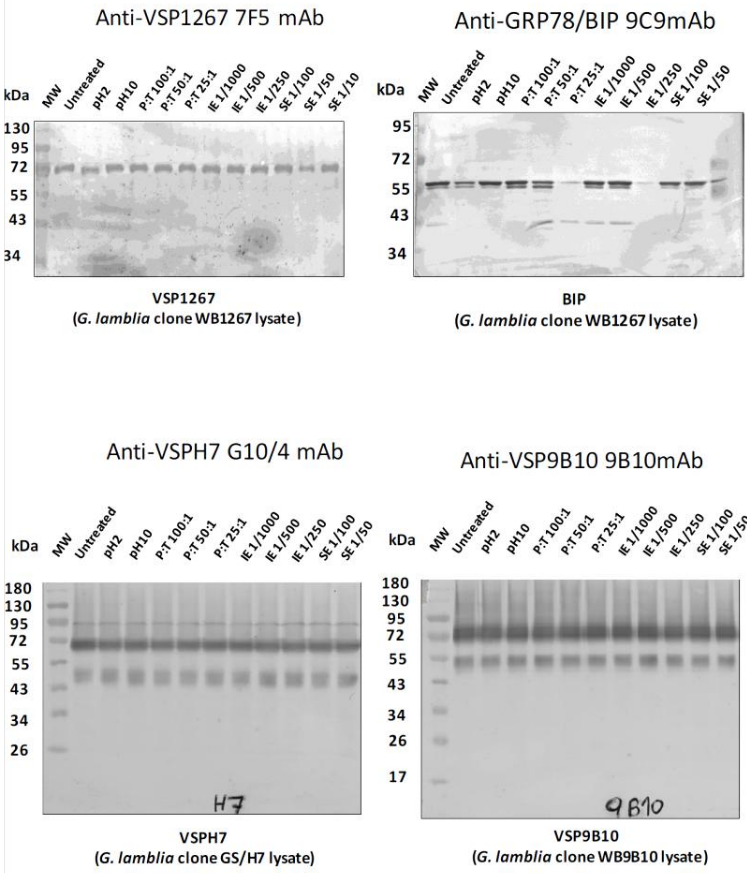

Figure 1e

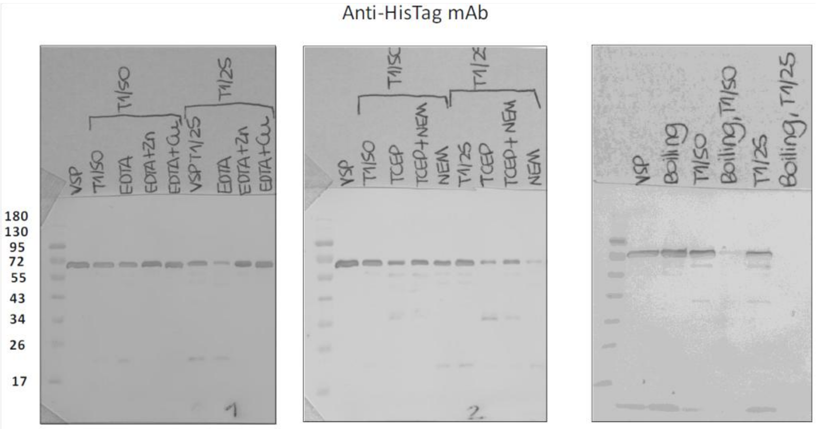

Figure 3c

Anti-HA mAb (clone 15E4)
Anti-NA rabbit pAb (I7649-48 USBiologicals)
Anti-VSP1267 mAb (clone 7F5)
Anti-GAG mAb (clone R187)

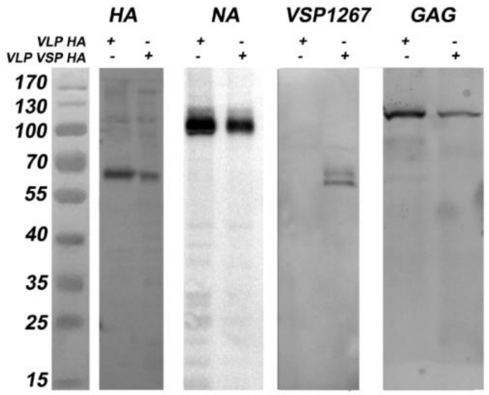

Figure 4a

Anti-VSP1267 7F5 mAb

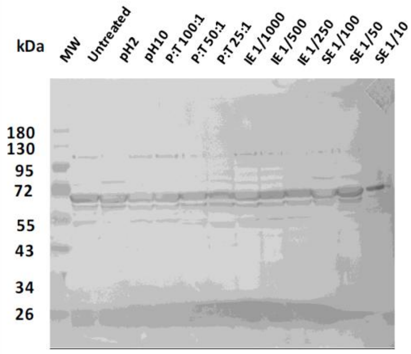

VSP1267
(from VLP-HA/VSP-G)

Anti-HA 15E4 mAb

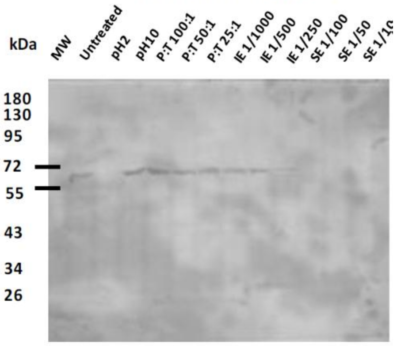

HA
(from VLP-HA)

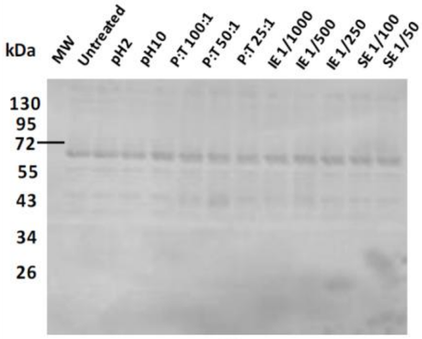

HA
(from VLP-HA/VSP-G)

Figure 4a

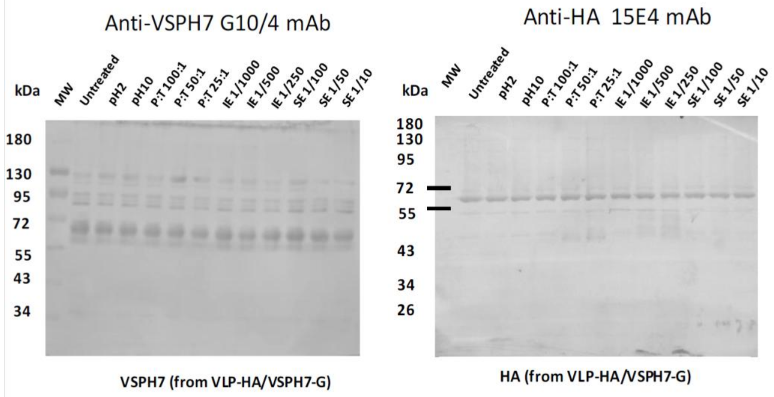

Anti-VSPH7 G10/4 mAb
kDa
MW
Untreated
pH2
pH10
P:T 100:1
P:T 50:1
P:T 25:1
IE 1/1000
IE 1/500
IE 1/250
SE 1/100
SE 1/50
SE 1/10
180
130
95
72
55
43
34
VSPH7 (from VLP-HA/VSPH7-G)
Anti-HA 15E4 mAb
kDa
MW
Untreated
pH2
pH10
P:T 100:1
P:T 50:1
P:T 25:1
IE 1/1000
IE 1/500
IE 1/250
SE 1/100
SE 1/50
SE 1/10
180
130
95
72
55
43
34
26
HA (from VLP-HA/VSPH7-G)
