# Supplementary material for: Efficient oral vaccination by bioengineering virus-like particles with protozoan surface proteins
Source: Nat Commun. 2019 Jan 21;10:361. doi: 10.1038/s41467-018-08265-9 (PMC6341118; doi:10.1038/s41467-018-08265-9)
Supplement: Supplementary file 3 — Reporting Summary [file 41467_2018_8265_MOESM3_ESM.pdf]

nature research

Corresponding author(s): Hugo D. Luján

Reporting Summary

Nature Research wishes to improve the reproducibility of the work that we publish. This form provides structure for consistency and transparency in reporting. For further information on Nature Research policies, see Authors & Referees and the Editorial Policy Checklist.

Statistical parameters

When statistical analyses are reported, confirm that the following items are present in the relevant location (e.g. figure legend, table legend, main text, or Methods section).

n/a	Confirmed	
☐	☒	The exact sample size (n) for each experimental group/condition, given as a discrete number and unit of measurement
☐	☒	An indication of whether measurements were taken from distinct samples or whether the same sample was measured repeatedly
☐	☒	The statistical test(s) used AND whether they are one- or two-sided<br>*Only common tests should be described solely by name; describe more complex techniques in the Methods section.*
☒	☐	A description of all covariates tested
☒	☐	A description of any assumptions or corrections, such as tests of normality and adjustment for multiple comparisons
☐	☒	A full description of the statistics including central tendency (e.g. means) or other basic estimates (e.g. regression coefficient) AND variation (e.g. standard deviation) or associated estimates of uncertainty (e.g. confidence intervals)
☐	☒	For null hypothesis testing, the test statistic (e.g. F, t, r) with confidence intervals, effect sizes, degrees of freedom and P value noted<br>*Give P values as exact values whenever suitable.*
☒	☐	For Bayesian analysis, information on the choice of priors and Markov chain Monte Carlo settings
☒	☐	For hierarchical and complex designs, identification of the appropriate level for tests and full reporting of outcomes
☒	☐	Estimates of effect sizes (e.g. Cohen's d, Pearson's r), indicating how they were calculated
☐	☒	Clearly defined error bars<br>*State explicitly what error bars represent (e.g. SD, SE, CI)*

Our web collection on statistics for biologists may be useful.

Software and code

Policy information about availability of computer code

Data collection	No software was used
Data analysis	Prism 5 version 5.0 (GraphPad Software) was used to perform statistical analysis.

For manuscripts utilizing custom algorithms or software that are central to the research but not yet described in published literature, software must be made available to editors/reviewers upon request. We strongly encourage code deposition in a community repository (e.g. GitHub). See the Nature Research guidelines for submitting code & software for further information.

Data

Policy information about availability of data

All manuscripts must include a data availability statement. This statement should provide the following information, where applicable:

- Accession codes, unique identifiers, or web links for publicly available datasets
- A list of figures that have associated raw data
- A description of any restrictions on data availability

The data associated with the paper has been deposited in a persistent repository with this identifier: DOI 10.17605/OSF.IO/9WDCA [https://osf.io/9WDCA/].

Field-specific reporting

Please select the best fit for your research. If you are not sure, read the appropriate sections before making your selection.

☒ Life sciences ☐ Behavioural & social sciences ☐ Ecological, evolutionary & environmental sciences

For a reference copy of the document with all sections, see nature.com/authors/policies/ReportingSummary-flat.pdf

Life sciences study design

All studies must disclose on these points even when the disclosure is negative.

Sample size	Sample sizes were chosen based on similar studies performed by us or found in the literature.
Data exclusions	No data were excluded from the analysis
Replication	All the experimental findings were reproducible and all the replicates obtained were included in the analysis
Randomization	For animal studies littermates of the same sex were randomly assigned to experimental groups
Blinding	Tumor volume determinations were carried out in a blinded fashion

Reporting for specific materials, systems and methods

Materials & experimental systems

n/a	Involved in the study	
☐	☒	Unique biological materials
☐	☒	Antibodies
☐	☒	Eukaryotic cell lines
☒	☐	Palaeontology
☐	☒	Animals and other organisms
☒	☐	Human research participants

Methods

n/a	Involved in the study	
☒	☐	ChIP-seq
☐	☒	Flow cytometry
☒	☐	MRI-based neuroimaging

Unique biological materials

Policy information about availability of materials

Obtaining unique materials	The biological samples from the animals used are not available since there were consumed in the analysis. The monoclonal antibodies genereted are available upon request.

Antibodies

Antibodies used	The details of the antibodies used are described in the methods section.
Validation	For the new monoclonal antibodies, the ELISA technique was used for the validation. In addition immunofluorscence using mAb 7F5 and western blotting using mAb 15E4 were carried out.

Eukaryotic cell lines

Policy information about cell lines

Cell line source(s)	The details of the cell lines used are described in the methods section
Authentication	None of the mammalian cell lines were authenticated after purchasing. Parasites were authenticated by PCR and sequencing of key genes.
Mycoplasma contamination	All cell lines were regularly tested and remained negative for Mycoplasma spp.

Commonly misidentified lines (See ICLAC register)	HEK cells were used.

Animals and other organisms

Policy information about studies involving animals; ARRIVE guidelines recommended for reporting animal research

Laboratory animals	A complete description of the animals used is included in the methods section
Wild animals	No wild animals were used.
Field-collected samples	No field-collected samples were used.

Flow Cytometry

Plots

Confirm that:

- [x] The axis labels state the marker and fluorochrome used (e.g. CD4-FITC).
- [x] The axis scales are clearly visible. Include numbers along axes only for bottom left plot of group (a 'group' is an analysis of identical markers).
- [x] All plots are contour plots with outliers or pseudocolor plots.
- [x] A numerical value for number of cells or percentage (with statistics) is provided.

Methodology

Sample preparation	The preparation of each sample is described in the methods section
Instrument	Accuri C6 Flow cytometer (BD)
Software	FlowJo vX.0.7
Cell population abundance	Not applicable
Gating strategy	The population of interest was gated on FSC-A vs SSC-A and then on FSC-A vs FSC-H to exclude doublets. For the dendritic cells experiments, the CD11c-PE+ cells were gated and analyzed. For the cytotoxicity experiments, the CFSE+ cells were gated and analyzed.

- [x] Tick this box to confirm that a figure exemplifying the gating strategy is provided in the Supplementary Information.
